# Evaluation of engineering properties for waste control of tomato during harvesting and postharvesting

**DOI:** 10.1002/fsn3.986

**Published:** 2019-03-10

**Authors:** Ahmad Jahanbakhshi, Vali Rasooli Sharabiani, Kobra Heidarbeigi, Mohammad Kaveh, Ebrahim Taghinezhad

**Affiliations:** ^1^ Department of Biosystems Engineering University of Mohaghegh Ardabili Ardabil Iran; ^2^ Department of Biosystems Engineering Ilam University Ilam Iran; ^3^ Moghan College of Agriculture and Natural Resources University of Mohaghegh Ardabili Ardabil Iran

**Keywords:** hydrodynamic properties, mechanical properties, nutritional properties, physical properties, tomato, waste control

## Abstract

In Iran, more than 30% of agricultural products turn into waste at different stages from harvesting to consumption. Thus, main factors for performing of this present study are including of: (a) the importance of tomato as an agricultural product and (b) lack of information about reducing waste during tomato processing. In this study, some physical, nutritional, mechanical, and hydrodynamic properties of tomato were measured under standard conditions. Physical properties included the length, width, thickness, mean diameter (geometric and arithmetic), mass, volume, density, sphericity, surface area, and aspect ratio. Also, nutritional properties, moisture, dry matter, pH, total soluble solid (TSS), and titration acidity (TA) of tomato were evaluated. The mechanical properties of tomato (compression and shear) were measured using Instron instrument. The hydrodynamic properties were measured with water in transportation, separation, and sorting of tomatoes. The physical properties were including of length, width, thickness, mass, volume, and geometric and arithmetic mean diameters showed a direct relationship with the size of tomatoes. Also, volumetric mass (density) had an inverse relation with tomato size. Yield point and shear force were obtained 51.27 and 22.20 N, respectively. The nutritional properties such as pH value, TSS, and TA were equal to 4.22, 22.23^ο^Brix, and 2%, respectively. The hydrodynamic properties of tomatoes such as the terminal velocity, the tomatoes' rise time in the water column, the buoyancy force, and the drag force were obtained to be equal to 0.05 m/s, 10.11 S, 0.52 N, and 0.17 N, respectively.

## 1 INTRODUCTION

Tomato is one of the most important horticultural products that is produced in different parts of Iran. Tomato fruit has the most waste as compared to other crops because tomato fruit comprises from 93% to 95% water and 5% to 7% dry matter. The waste during the harvest and postharvesting of tomato were reported to be between 30% and 40% (Jahanbakhshi & Kheiralipour, 2019b; Mansori alam & Ahmadi, 2017; Shahroudi, Golmohammadi, & Kalanatri, 2018). In this regard, researchers have reported that there is a linear relationship between tomato's mechanical properties and its vulnerability with a 78% coefficient of determination (Desmet et al., 2004).

Researchers have always tried to determine the physical and mechanical properties of agricultural products as a basis for designing and building machines and equipment that they can be used in transportation, sorting, processing, and storage of agricultural products in order to obtain high‐quality products. The most important physical properties of agricultural products include characteristic of dimensions, mass, volume, surface area, image surface, coefficient of sphericity, aspect ratio, porosity, and static friction coefficient. The most important mechanical properties include yield force, deformation, hardness, and breakdown energy (Işıklı, Şenol, & Çoksöyler, 2014; Jahanbakhshi, 2018; Jahanbakhshi, Abbaspour‐Gilandeh, & Gundoshmian, 2018; Jahanbakhshi & Kheiralipour, 2019a; Yıldız, İzli, Ünal, & Uylaşer, 2015). Wastage rate in agricultural products will increase directly or indirectly by mechanical damage. Bruising caused by a quasi‐static force is one of the most important reasons for lower quality in fresh fruits. At different stages of harvesting and postharvesting such as transfer, transportation, storage, and processing, agricultural products are exposed to numerous mechanical forces and physical damage. In many cases, the forces cause the mechanical damage on the product and tear its cell wall. This topic has become one of the most important issues for modeling and experimental analysis in the field of biosystems engineering (Grotte, Duprat, Loonis, & Pietri, 2001; Jahanbakhshi & Kheiralipour, 2019b; Masoudi, Tabatabaeefar, & Borghei, 2006; Myhan, Białobrzewski, & Markowski, 2012; Salehi, Taghizadeh‐Alisaraei, Jahanbakhshi, & Shahidi, 2018; Salehi, Taghizadeh‐Alisaraei, Shahidi, & Jahanbakhshi, 2018).

A fruit falls into a liquid with a constant speed, when resultant of vertically force on sample to be equal to zero. This speed is called terminal velocity (Jordan & Clark, 2004; Kheiralipour, Tabatabaeefar, Mobli, Mohtasebi et al., 2010; Mirzaee et al., 2008). In the hydraulic transfer of fruits, the liquid speed is determined by two factors of fruit density and shape and, thus, difference in fruits quality can be recognized by differences in density (Lorestani & Ghari, 2012; Lorestani & Tabatabaeefar, 2006; Tabatabaeefar & Rajabipour, 2005). The terminal velocity of a fruit that moves in a liquid with a higher or a lower density than that of the fruit can be considered as an appropriate method for separating fruits. Thus, fruits with different terminal velocities pass certain distances in the duct to reach different depths. Therefore, samples can be separated using a suitable separator under its terminal velocity (Jordan & Clark, 2004).

So far, many studies have been carried out in the world on determining the physical, nutritional, mechanical, and hydrodynamic properties of different agricultural products. Some of those studies are referred to in the following.

The volume and density of agricultural products are very important in different processes and in the assessment of product quality such as determination of fruit ripening (Sitkei, 1987). Jaliliantabar, Lorestani, and Gholami (2013) investigated the physical properties of the Kumquat fruit. They reported the values of 14.3 g, 12.3 ml, 29.4 mm, 2,743 mm^2^, and 74.5% for mean mass, volume, geometric mean diameter, surface area, and sphericity, respectively. Moghadam and Kheiralipour (2015) conducted a research on the physical and nutritional properties of the hawthorn fruit. They reported that some physical properties such as sphericity, surface area, and slenderness ratio were 1.13%, 1.69 mm, and 1.26, respectively. After studying the nutritional properties of the hawthorn fruit, they concluded that the TSS and TA were equal to 18.7% and 1.71%, respectively. Jahanbakhshi, Yeganeh, and Akhoundzadeh Yamchi (2016) studied the physical, mechanical, and hydrodynamic properties of scolymus. They reported that the scolymus density is less than water, and thus, it could be hydraulically sorted and transferred without any damage. The maximum force for bending and shearing the scolymus were 41.5 and 82.9 N, respectively. Moreover, in the assessment of hydrodynamic properties, the average terminal velocity for the scolymus was equal to 0.02 m/s.

In investigation of the physical and mechanical properties of snake melon, Jahanbakhshi (2018) reported that length, width, thickness, surface area, and density are some of the physical characteristics that play an important role in many of the topics related to designing special machines or assessing materials' behavior when they are transferred. The maximum force for the pressure, bending, and shearing tests on the snake melon were 309.66, 44.4, and 33.66 N, respectively. Singh and Reddy (2006) conducted a study about the postharvest mechanical properties of orange peel and fruit. They measured the shearing energy of the orange fruit and showed that increase in the storage period would reduce the amounts of force and energy required to shear oranges. Ince, Uğurluay, Güzel, and Özcan (2005) carried out a study on the flexural and shear properties of sunflower. They found out that increasing of moisture was caused to reduce shear modulus of elasticity and bending stress and increase shear energy. Kheiralipour (2008) studied the terminal velocity of two apple cultivars and reported that the fruits reached their terminal velocity at 5 s after release. Terminal velocity of fruits and Cereals small has an important role in designing the equipment for transportation of materials through wind or water, designing of fluidized bed dryers. Thus, researchers determined terminal velocity for different products such as pistachios and green peas (Kashaninejad & Tabil, 2009; Nimkar & Chattopadhyay, 2002).

Tomato is one of the plants which are sensitive to environmental stresses including of intensive temperature, high salinity, dryness, and environmental pollution. Therefore, according to different environmental stresses in different regions of Iran, various cultivars are planted in different areas. In Kermanshah province, *tompler cultivar* of tomato has been considered by farmers. However, due to lack of knowledge about the engineering properties of this product, its waste during the process of harvesting and after that is a matter worthy of attention and analysis. So, the importance of tomato as an agricultural product and lack of knowledge among Iranian farmers about how to reduce waste and process tomatoes are factors that motivate this study. Review of the previous literature revealed that prior studies had not researched the properties of this cultivar of tomatoes.

## 2 MATERIALS AND METHODS

### 2.1 Determination of physical properties

In this study, the tomatoes which had equal rates of ripeness were collected from a farm in Kermanshah province in Iran as the sample. Then, in order to prevent the initial moisture of the product, the sample was kept inside a refrigerator at the temperature of 4 ± 1°C and transferred from the storage environment (the refrigerator) to the laboratory about 2 hr before carrying out the tests. To determine the dimensions and mass of the samples under experiment, a digital caliper with the precision of 0.01 mm and a digital scale with the accuracy of 0.01 g were used. Geometric mean diameter (*D*_g_), arithmetic mean diameter (*D*_a_), and sphericity percentage (Ø) were calculated through Equations (1), (2), (3) (Mohsenin, 1986).

(1) $$D_{g}=\sqrt[{3}]{\mathrm{LWT}}$$

(2) $$D_{a}=\frac{L+W+T}{3}$$

(3) $$\phi =\frac{D_{g}}{L}$$

where *L*,* W,* and *T* were the length, width, and thickness of the tomatoes, respectively. *S* is the surface area (mm^2^), and *R*_a_ is the aspect ratio of the tomato were obtained through Equations (4) and (5) (Mohsenin, 1986).

(4) $$S=\pi D_{g}^{2}$$

(5) $$R_{a}=\frac{W}{L}$$

The tomatoes mass was measured using a digital scale (GF600, USA). To determine the volume of the tomatoes, the platform method was used (Equation (6)) (Mohsenin, 1986).

(6) $$V=\frac{W_{W}}{\rho_{W}}$$

The density of the tomatoes is obtained by Equation (7).

(7) $$\rho_{t}=\frac{M}{V}$$

where *W*_W_ is the density of the displaced water (g/cm^3^), *ρ*_W_ is the density of the water (g/cm^3^), *ρ*_t_ is the true density (g/cm^3^), *M* is the mass (g), and *V* is the volume of the tomatoes (m^3^).

Static friction coefficient (*μ*_s_) of tomatoes was calculated by measuring the angle at which tomatoes started moving on four surfaces of galvanized, aluminum, wood, and rubber sheets. For measurement of this parameter, a metal rectangular cube whose both ends were open with the dimensions of 20 × 10 × 10 cm was placed on the given surface and filled with tomatoes. After that, the gradient of the surface under study was gradually increased and the rectangular cube started to move at a particular angle without being in contact with the surface. At that point, the tangent of the angle between the surface and the horizon (*α*) was taken as the static friction coefficient calculated through Equation (8) (Khazaei, Borghei, & Rasekh, 2003; Mohsenin, 1986).

(8) $$\mu_{s}=\tan\left(\alpha \right)$$

### 2.2 Determination of nutritional properties

Measurement of moisture content was done using the standard oven hot air method (Memmert UNE 500 model). For this purpose, 20 g samples of tomatoes were dried at an oven for 4 hr at the temperature of 105°C in three replications. Weight of samples was measured before and after being placed in the oven using a digital scale. Then, the moisture content and dry matter of tomato fruit were calculated by Equations (9) and (10), respectively (Jahanbakhshi et al., 2018; Kaveh, Jahanbakhshi, Abbaspour‐Gilandeh, Taghinezhad, & Moghimi, 2018):

(9) $$\text{MC}=\frac{M_{W}-M_{d}}{M_{W}}$$

(10) $$\text{DM}=\frac{M_{d}}{M_{W}}\times 100$$

Where MC is the moisture content of fruit (%), *M*_W_ is the initial mass of fruit (g), *M*_d_ is the mass of dried fruit (g), and DM is the dry matter fruit (%).

The pH of tomato juice was measured by pH meter (pH‐200L model). The TA was measured using the titration method. 0.1 normal soda (NaOH) (5ml of tomato extract in 50 ml of distilled water) and the titration was operated until solution pH reached 8.2. The results were expressed as grams of malic acid per 100 g fresh weight. The TSS was measured for 10 tomatoes in Brix degrees using a refractometer instrument (model ATC‐le manual model). Equations (11) and (12) were used to measure the TA and $\frac{\text{TSS}}{\text{TA}}$ ratio.

(11) $$\text{TA}=\left[\frac{N_{n}V_{b}E}{V_{i}}\right]\times 100\times 0.001$$

where TA is the titratable acidity (%), *V*_b_ is the amount of soda in milliliters used for titration, *N*_n_ is the normality of the soda consumed (*N*_n_ = 0.1), *E* is the equivalent gram of the dominant acid, and *V_i_* is the volume of the sample tomato extract in milliliters.

(12) $$T=\frac{\text{TSS}}{\text{TA}}$$

where *T* is the $\frac{\text{TSS}}{\text{TA}}$ ratio, TSS is the total soluble solids (°Brix), and TA is the titratable acidity (%).

### 2.3 Determination of mechanical properties

**Figure 1:**
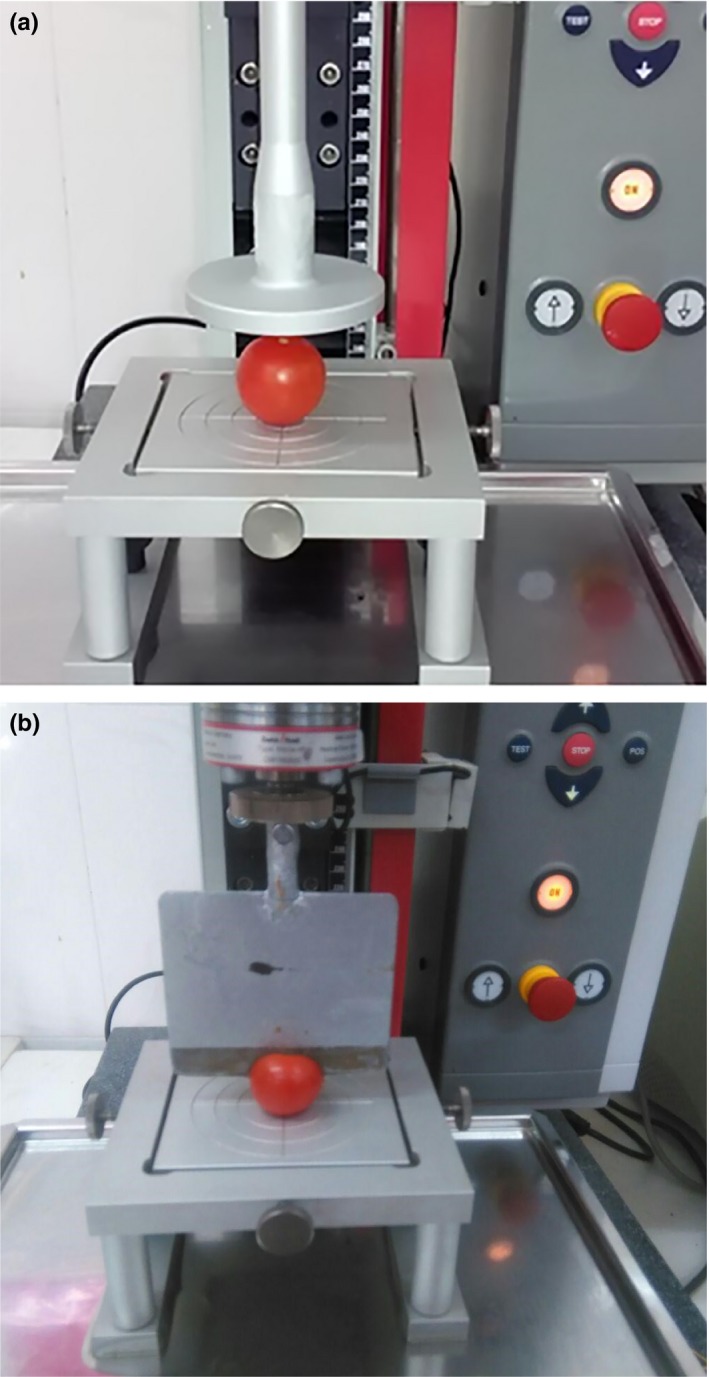
(a) Compression test and (b) Shear test

The mechanical properties of tomato fruit were including of compression and shear test. This test was performed using Instron machine (Z 0.5 model, country Germany). For compression test, the samples were placed on the flat plate and pressed with a movable plate and a 500 N load cell fixed parallel to the base. For the shear test, a straight edge blade with the thickness of 1.4 mm and the blade angle of 30 degrees was used based on the DIN 53294 standard. The mechanical test was conducted at room temperature and cross‐head speed of 20 mm/min (Jahanbakhshi, 2018; Jahanbakhshi & Kheiralipour, 2019b). The Instron machine was simultaneously connected to a computer, and data mining was carried out (Figure 1).

### 2.4 Determination of hydrodynamic properties

**Figure 2:**
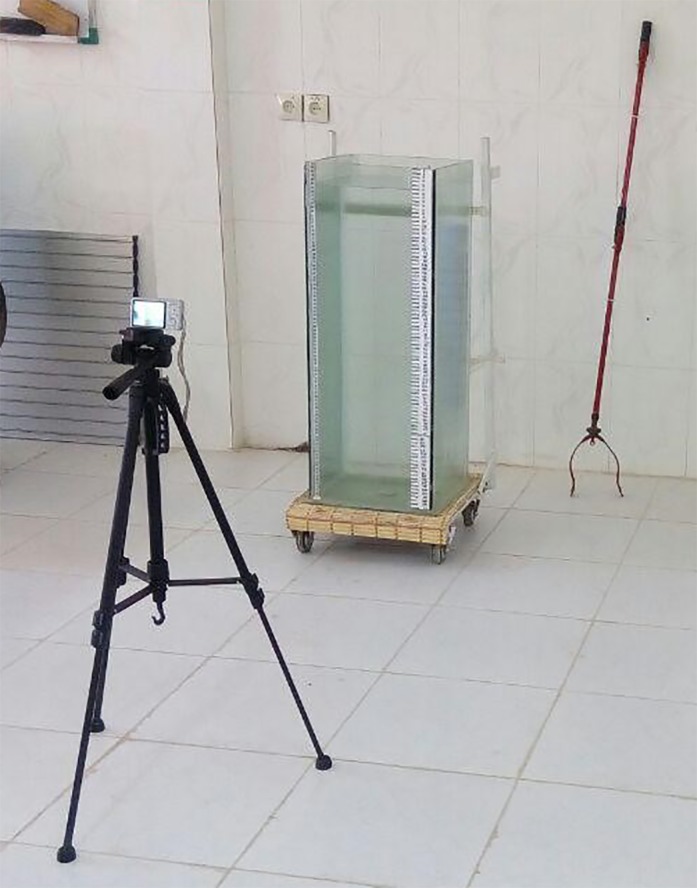
Plexiglas column, the clamp, and the position of the camera

In determining, the hydrodynamic properties were measured in a plexiglas column with the base of 35 × 35 × 90 cm and the thickness of 8 mm. The sides of this column's base were set to fit standard sizes (Jahanbakhshi et al., 2016; Kheiralipour & Marzbani, 2016; Vanoni, 2006). The column was filled with water up to 80 cm of its height. Each tomato was placed on bottom of water by a nondestructive clamp. It was then released after the water became calm. Immediately, a Sony (DSC‐W710) digital camera was used to film the movement of each tomato from the beginning to the end with 30 frames per second (Figure 2).

**Figure 3:**
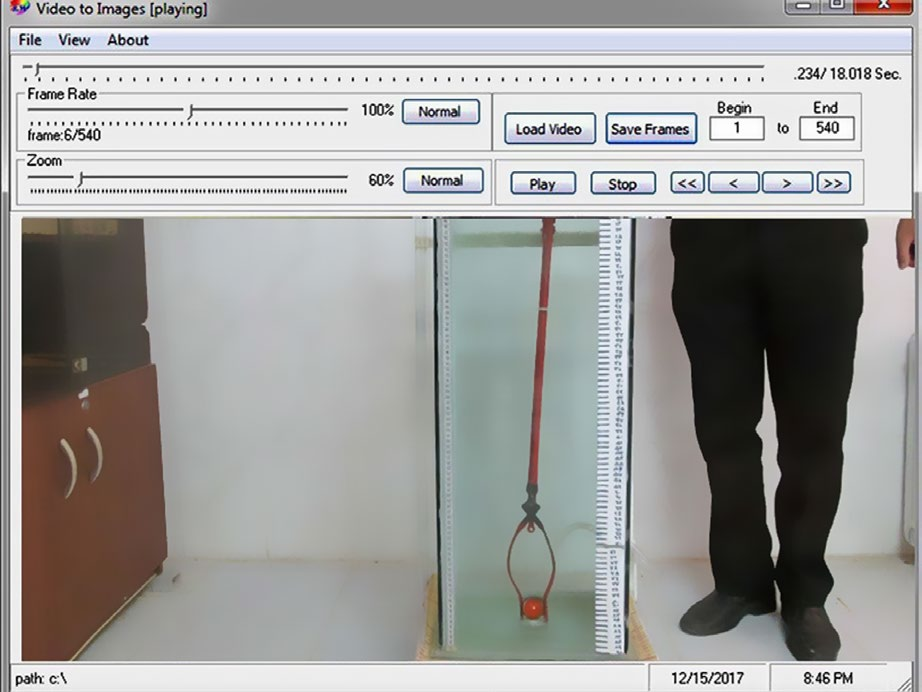
A view of the VCI.exe software

**Figure 4:**
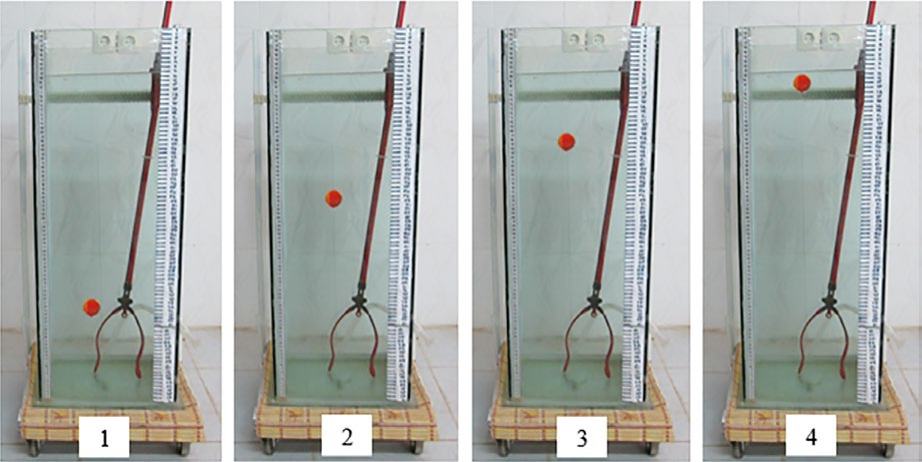
Moving the tomato in the water column

The film of each tomato's movement was converted to images (Figures 3 and 4) using a video conversion to image software (VCI.exe). The camera that was used can record 30 images per second. Thus, each image takes place in 0.033 S. The time for each tomato's rise to the height of 80 cm was multiplied by the number of shots per move in 0.033 S. Since little time is required for tomatoes to reach their maximum speed (terminal velocity) from zero, to calculate terminal velocity for the first 30 cm, the movement of the tomatoes was ignored and thus the 50 cm vertical distance (80–30) was taken as the vertical path for tomatoes' movement. The terminal velocity of each tomato (*V*_t_) was calculated through Equation (13) (Jahanbakhshi et al., 2016).

(13) $$V_{t}=\frac{50\times 10^{-2}}{\left(0.033\;\times \;N\right)}$$

In that relation, *N* is the number of images for a tomato's movement at the vertical distance of 50 cm. *F*_b_ is the buoyancy force or the Archimedes force that affected the tomatoes in the opposite direction of their weight force. This force is obtained using Equation (14) (Jahanbakhshi et al., 2016; Kheiralipour, Tabatabaeefar, Mobli, Mohtasebi et al., 2010; Kheiralipour et al., 2008).

(14) $$F_{b}=\rho_{w}Vg$$

where *V* is the volume of the tomato (m^3^), *g* is the acceleration of gravity (m/s^2^), and *ρ*_w_ is the density of water (Kg/m^3^). The drag force (*F*_d_) is the force on the tomato in the opposite direction of its movement. Equation (15) was used to obtain this drag force. This relation clearly shows that when a tomato is in a static state, no drag force affects it but as soon as it starts moving, the drag force rises from zero, and when the tomato reaches its terminal velocity, the drag force reaches its maximum that equals:

(15) $$F_{d}=0.5\rho_{w}V_{t}^{2}C_{D}A$$

Where *ρ*_w_ is the water's density (Kg/m^3^), *V*_t_ is the tomato's terminal velocity (m/s), *C*_D_ is the drag coefficient, and *A* is the surface area of tomato (m^2^). In Equation (15), *C*_D_ is the drag coefficient and is a function of the fruit's velocity. Applying the Stoke's law at low velocities (*N*_R_ < 1), we will obtain (Crowe, Elger, & Roberson, 2001):

(16) $$C_{D}=\frac{24}{N_{R}}$$

and

(17) $$N_{R}=\frac{VD\rho_{w}}{\mu_{w}}$$

and then

(18) $$C_{D}=\frac{24\mu_{w}}{VD\rho_{w}}$$

where in *N*_R_ is the Reynolds number (without any dimensions), *µ*_w_ is the static water viscosity as a function of temperature. Here, water temperature is assumed to be constant. *D* is the diameter of a tomato (m).

## 3 RESULTS AND DISCUSSION

### 3.1 Physical properties

The physical properties of tomato are reported in Table 1. The mean length, width, thickness, arithmetic mean diameter, geometric mean diameter, surface area, sphericity, aspect ratio, mass, volume, and true density of the tomato were 33.46, 36.04, 34.84, 34.78, 34.75 mm, 3,810.04 mm^2^, 0.98%, 1.07, 24.76 g, 51.62 cm^3^, and 0.47 g/cm^3^, respectively. Improper packaging and insufficient transportation are part of the agricultural products' wastes. Weight, size, and shape of agricultural products are the most important parameters that are used to reduce waste during packaging and transportation. In the design of transmission systems, grading and cleaning the product, sphericity and true density are of particular importance, which in this study were 0.98% and 0.47 g/cm^3^, respectively. In the hydraulic transportation of agricultural products, product density and shape are considered as two important factors. As the density of tomato (0.47 g/cm^3^) is low as compared to water, it can be washed using the flow of water. The importance of these properties for the design of harvesting machines, separation, and transportation in order to control waste losses had been emphasized by other researchers such as Jahanbakhshi et al. (2018), Jahanbakhshi et al. (2016), Kabas, Ozmerzi, and Akinci (2006), Kheiralipour, Tabatabaeefar, Mobli, Mohtasebi et al. (2010), Moghadam and Kheiralipour (2015) and Rafiee et al. (2007).

**Table 1:** Physical properties of tomato fruit

Parameters	Mean	Max	Min	*SD*	CV %
Length (mm)	33.46	37.75	27.74	2.65	7.91
Width (mm)	36.04	40.63	30.39	2.33	6.46
Thickness (mm)	34.84	39.04	28.94	2.33	6.68
Arithmetic mean diameter (mm)	34.78	38.93	29.20	2.29	6.58
Geometric mean diameter (mm)	34.75	38.92	29.18	2.30	6.61
Surface area (mm^2^)	3,810.04	4,756.92	2,674.85	496.76	13.03
Sphericity (%)	0.98	1.04	0.96	0.03	3.06
Aspect ratio	1.07	1.19	0.98	0.05	4.67
Mass (g)	24.76	36.20	14.53	4.87	19.66
Volume (cm^3^)	51.62	68	37	6.28	12.16
True density (g/cm^3^)	0.47	0.64	0.36	0.05	10.63

The static friction coefficients for four surfaces of galvanized, aluminum, wood, and rubber sheets were calculated, and the results were reported in Table 2. The average static friction coefficients on the different surfaces stated above were 0.249, 0.212, 0.364, and 0.305, respectively. These static friction coefficients show that tomatoes have the lowest friction on the surface of aluminum sheets (0.212). This rate is significantly lower than the rates obtained in the other treatments, and a lower gradient angle is required for the transfer of tomatoes. Also, the highest static friction coefficient is for wood surface (0.364) and this shows that the adhesion of this tomato variety to the wood surface. Therefore, for reduction of waste, it is suggested that aluminum surfaces are used during the tomato transfer. In similar studies, researchers reported that the value of friction coefficient of agricultural products is very important in the design of harvesting, processing, transportation, and storage equipment (Asgarian Najaf Abadi, Ghasemzadeh, & Hajiloo, 2015; Askari Asli‐Ardeh, Mohammad Zadeh, & Abbaspour‐Gilandeh, 2017; Ganji, Rajabipoor, & Alimardani, 2011; Kabas et al., 2006; Topuz, Topakci, Canakci, Akinci, & Ozdemir, 2005).

**Table 2:** Static frictional coefficient of tomato fruit

Surface type	Mean	Max	Min	*SD*	CV %
Galvanized iron steel	0.249	0.286	0.221	0.020	8.33
Aluminum	0.212	0.249	0.194	0.018	4.76
Wood	0.364	0.404	0.338	0.020	5.55
Rubber	0.305	0.344	0.284	0.019	3.33

### 3.2 Nutritional properties

The nutritional properties of the tomato fruit are reported in Table 3. The means for the moisture content and the dry matter of tomato fruit were 94.29 and 5.70 percent. In similar studies, researchers reported a moisture content is very important and influential in determination of physical and mechanical properties of fruits (Altuntaş & Yıldız, 2007; Gholmohammadi, Roghanipour, & Mesri Ghendishmin, 2013; Moghadam & Kheiralipour, 2015). The average pH, TSS, TA for the tomato juice were 4.22 and 22.23^ο^Brix and 2%, respectively. In addition, these data were used to obtain the TSS/TA ratio which was equal to 11.13. The properties mentioned above are very important in preserving the product after harvest, during storage and processing. A similar study conducted by Kheiralipour (2008). He reported the pH values for two apple cultivars, namely Red Spark (3.91) and Delbar Stival (3.61). The present study shows that the pH value for tomato (4.22) is significantly higher than those of the apple cultivars mentioned. In addition, the average TSS for the two apple cultivars was 10.73 and 12.54^ο^Brix, respectively. According to this study, tomato has higher TSS (22.23^ο^Brix) as compared to those apple cultivars.

**Table 3:** Nutritional properties of tomato fruit

Parameters	Mean	Max	Min	*SD*	CV %
Moisture content (%)	94.29	94.68	93.67	0.31	0.32
Dry matter (%)	5.70	6.33	5.31	0.31	5.43
pH	4.22	4.40	3.87	0.19	4.50
Total soluble solid (TSS, ^ο^Brix)	22.23	22.89	21.89	0.30	1.34
Titratable acidity (%)	2.00	2.20	1.83	0.11	5.50
TSS/TA ratio	11.13	11.97	11.11	0.62	5.57

### 3.3 Mechanical properties

The mechanical properties of the tomato fruit for compression test are reported in Table 4. The mean values of the properties measured in the compression test (elasticity module, the maximum force required [rupture force], deformation, energy) were 0.11 GPa, 51.27 N, 9.52 mm, and 202.38 N·mm, respectively. One of the most important factors in increasing agricultural waste is mechanical damage. Therefore, in order to reduce tomato waste, the compressive forces caused by displacement and transportation are reduced to the lowest possible (<51.27 N). In a similar study, Jahanbakhshi and Ghamari (2015) investigated the mechanical properties of plum fruit. In a compression test, they reported the modulus of elasticity equal to 0.0118 GPa. As compared to the present study, it can be stated that tomato fruit has a higher modulus of elasticity (0.11 GPa) and, thus, it has a harder tissue.

**Table 4:** Mechanical properties of tomato fruit in the pressure test

Parameters	Mean	Max	Min	*SD*	CV %
Elasticity modulus (GPa)	0.11	0.14	0.07	0.03	27.27
*F*_max_ (N)	51.27	74.80	34.30	17.08	33.31
DL at *F*_max_ (mm)	9.52	11.80	8.60	1.52	15.96
W to *F*_max_ (N·mm)	202.38	350.64	115.73	70.32	34.74

Another mechanical property of the tomato fruit is the shear force that it is shown in Table 5. The mean values of the measured properties in the shear test were including of Shear modulus, maximum force required for shearing tomato fruit, shear strength, and shear deformation, that their amounts were 0.059 N/mm^2^, 22.20 N, 0.017 N/mm^2^, and 12.73 mm, respectively. These characteristics can be useful for waste control and tomato processing in factories. In similar study, results of this research are in agreement with reports of Jahanbakhshi and Ghamari (2015). They reported the average shear modulus and shear force of 0.088 N/mm^2^ and 23.70 N, respectively.

**Table 5:** Mechanical properties of tomato fruit in the shear test

Parameters	Mean	Max	Min	*SD*	CV %
Shear modulus (N/mm^2^)	0.059	0.076	0.050	0.011	18.64
*F*_B_ (N)	22.20	32.10	17.40	6.70	30.18
*T*_B_ (N/mm^2^)	0.017	0.020	0.015	0.002	11.76
*ν*_B_ (mm)	12.73	16.56	8.93	3.21	25.21

### 3.4 Hydrodynamic properties

The hydrodynamic properties of tomato fruit are reported in Table 6. The mean terminal velocity, climb time for tomato in column, buoyancy force, and drag force were 0.05 m/s, 10.11 S, 0.52 N, and 0.17 N, respectively. The density of tomato (0.47 g/cm^3^) is lower than water. Thus, tomato rises in water and its terminal velocity is topside. So, it floats on top of water. Terminal velocity can be considered as a criterion for sorting tomatoes based on their sizes and cultivars. Tomatoes can be hydraulically washed, transported, or even wiped from impurities such as sand and the like without being at all damaged. In similar studies, researchers had reported that hydrodynamic properties of agricultural products are necessary for reduction of their waste during transportation, separation, and sorting by water (Jahanbakhshi et al., 2016; Kheiralipour, Tabatabaeefar, Mobli, Mohtasebi et al., 2010; Kheiralipour et al., 2008). Taking other parameters into consideration, it was observed that the terminal velocity of each tomato is among others more affected by density so that increase in density would lead to decrease in terminal velocity. Other similar studies investigated terminal velocity and rise time for two apple cultivars, Redspar and Delbarstival, and modeled terminal velocity of kiwi fruit. Their results indicated that reducing the real density would increase the termination velocity (Kheiralipour, 2008; Kheiralipour, Tabatabaeefar, Mobli, Rafiee et al., 2010).

**Table 6:** Hydrodynamic properties of tomato fruit

Parameters	Mean	Max	Min	*SD*	CV %
Terminal velocity (m/s)	0.05	0.07	0.04	0.007	14.00
Rise time tomato in the water column (S)	10.11	12.40	6.30	1.33	13.15
Buoyancy force (N)	0.52	0.66	0.36	0.06	11.53
Buoyancy force (N)	0.17	0.32	0.12	0.03	17.64

## 4 CONCLUSION

The first step in the codification of quality standards for agricultural products such as tomatoes as well as the improvement of different processing lines for this product is to know various properties of those products and their changes according to different factors. This study investigated some physical, nutritional, mechanical, and hydrodynamic properties of tomato. The following findings and results could be reached at based on the present study:

1. Tomatoes have high sphericity (0.98%). These features must be taken into account in designing transfer, displacement, and grading systems.
2. Due to low density of tomato (0.47 g/cm^3^) as compared to water, this product can be sorted, transported, and washed through water flow.
3. The maximum static friction coefficient was on a wood surface and equaled 0.364, and the minimum was on an aluminum surface and equaled 0.212. This result seems totally logical since a wood surface is the roughest, and the aluminum surface is the smoothest among the surfaces under investigation.
4. In studying the nutritional properties of tomato, the average pH, TSS, TA obtained from tomato juice were 4.22, 22.23^ο^Brix, and 2%, respectively.
5. For mechanical properties, the maximum force for compression and shear tests of the tomato fruit were 51.27 and 22.20 N, respectively.

## CONFLICT OF INTEREST

The authors have declared no conflict of interest.

## ETHICAL REVIEW

This study does not involve any human or animal testing.
